# Synchronous Telephone-Based Consultations in Teledentistry: Preliminary Experience of the Telehealth Brazil Platform

**DOI:** 10.1089/tmr.2020.0007

**Published:** 2021-01-08

**Authors:** Michelle Roxo-Gonçalves, Vanessa Müller Stüermer, Laura Ferraz dos Santos, Daniela dal Forno Kinalski, Elise Botteselle de Oliveira, Rudi Roman, Vinicius Coelho Carrard

**Affiliations:** ^1^ Oral Pathology Department, School of Dentistry, Universidade Federal do Rio Grande do Sul, Porto Alegre, Rio Grande do Sul, Brazil.; ^2^ TelessaudeRS-UFRGS, Federal Universidade Federal do Rio Grande do Sul, Porto Alegre, Rio Grande do Sul, Brazil.; ^3^ Postgraduate Programme in Epidemiology, School of Medicine, Universidade Federal do Rio Grande do Sul, Porto Alegre, Rio Grande do Sul, Brazil.; ^4^ Department of Oral Medicine, Hospital de Clínicas de Porto Alegre (HCPA), Porto Alegre, Rio Grande do Sul, Brazil.

**Keywords:** teledentistry, oral health, public health, primary health care

## Abstract

**Background:**

The Brazilian National Health System (NHS) has incorporated telehealth to improve the quality of care in recent decades. Among the actions taken, teleconsultations have been offered to support diagnosis and decision-making for health professionals.

**Methods:**

This cross-sectional study aimed to summarize the preliminary experience of a telephone-first consultation for oral health issues available for dentists and physicians from primary health care (PHC).

**Results:**

The study sample was 385 teleconsultations with oral health questions requested from all Brazil sent from May 2018 to July 2019, majority by dentists 83.2% (*n* = 332). Oral medicine was the main reason for teleconsultation (50.9%). Resolution in PHC was considered possible in 57.1% of cases (*n* = 220).

**Conclusions:**

It was concluded that a telephone-first consultation may be useful to improve the resolvability and the quality of care in the PHC on oral health issues. The teledentistry allows the resolution of oral issues in PHC, avoiding the displacement of patients to more distant specialized centers. Teledentistry could be more useful in the actual coronavirus disease (Covid-19) pandemic.

## Introduction

Information and Communication Technology (ICT) was inserted into the field of health to strengthen the quality of care, efficiency in the management of health uses, and the intelligent use of available information. Telehealth involves the use of ICT to support professionals who work in places where access to health services is a critical factor.^1–3^

Among the applications of telehealth is teleconsultation, which involves interventions that show the potential to overcome barriers to health care and information, especially to remote places. This sort of assistance can be offered asynchronously or synchronously, the first being in text form and the second in real time by video or phone call. Teleconsultation has been applied in some countries, mainly in medical specialties such as oncology, trauma, neurosurgery, dermatology, and psychiatry. In dentistry and nursing, these approaches have been less explored, but some experiences have already been reported.^4–11^

Brazil is a country with some gaps in the National Health System (NHS), such as the quality of the service provided, which is heterogeneous in the different levels of care.^12,13^ In this regard, the Brazilian Ministry of Health instituted the Brazilian Telemedicine Programme (www.telessaudebrasil.org.br) in 2007. This programme was created with the objective of strengthening and improving the quality of care in primary health care (PHC) with tools for permanent education and interprofessional communication through ICT. Currently, there are 26 telehealth centers distributed across 23 out of 27 Brazilian states.^14^

Among these centers is the Technical and Scientific Centre for Telehealth of Federal University of Rio Grande do Sul (TelessaúdeRS-UFRGS http://www.saude.gov.br/telessaude/nucleos-de-telessaude),^12^ a project developed through a partnership between the Ministry of Health and Federal University of Rio Grande do Sul. Since 2007, this center has been promoting teleconsultation, telediagnosis, and tele-education for professionals working in PHC services to improve the quality of care in the NHS. Among its particularities, TelessaúdeRS-UFRGS has a teleconsultation service offered through free-of-charge telephone calls. Through this service, PHC professionals from all over the country can ask questions to specialist teleconsultants.

This contact provides an opportunity for interprofessional discussion of clinical cases based on available scientific evidence, to support management and conduct, in addition to answering general questions of daily practice in an effective manner. This support allows cases that previously would be referred for face-to-face consultation with a specialist to be solved in the PHC, avoiding unnecessary travel and overcrowding of other levels of care.^5,15–17^ The teledentistry can have even more advantages during the current world emergency condition due to the Covid-19 dissemination.^18,19^ By offering this service, TelessaúdeRS-UFRGS generated a reduction in medical referrals, from 190,000 to 68,000, in different areas of health in Rio Grande do Sul, the southern Brazilian State, in a period of 2 years.^12,13^

The term teledentistry is used to refer to the inclusion of oral health care and education supported by ICT,^20^ often enabling support in care and cost reduction for the public health system, thus improving care results.^21^ Bavaresco et al.^4^ showed that offering synchronous and asynchronous teleconsultations (Skype™ call and text) to oral health teams induced a 45% reduction in referrals to specialized services from 2007 to 2012. In addition, the telediagnosis service for oral lesions (asynchronous teleconsultations) at TelessaúdeRS-UFRGS showed a reduction in the intention to refer from primary to specialized care (96.9–35.1%).^5^

Among the professional teleconsultants in the synchronous teleconsultation service of TelessaúdeRS-UFRGS, there are specialist dentists. They answer questions in real time and support decision-making, based on evidence, to applicants through telephone call. When the dentist calls, if necessary, the case can be discussed with other professionals in TelessaúdeRS such as physicians, nurses, and nutritionists (www.ufrgs.br/telessauders). The aim of this study was to describe a recent experience of the Telehealth Network of Rio Grande do Sul State with a telephone consultation for oral health issues.

## Methodology

This is a retrospective observational study. This study was approved by the local ethics committee (GPPG, Hospital de Clínicas de Porto Alegre, protocol 3.796.755). The variables of interesting were professional category of the applicants, gender, location (city and state) of origin of the requests, and the health area involved in the discussion, based on the diagnostic hypothesis. The data used in this study were retrieved from a web-based system developed by TelessaúdeRS-UFRGS. All synchronous telephone consultations related to oral health carried out by TelessaúdeRS from May 2018 to July 2019 were analyzed.

The teleconsultations were performed by free-of-charge telephone calls and originated from any city in the country. The service is available during the business hours of the basic health units (Monday–Friday, from 8 am to 8 pm). The first telephone contact of the public health professional (physicians or dentist) is with the TelessaúdeRS-UFRGS' attendants. Those professionals were responsible for verifying the professional's enrollment in the public health care system. After this, the call is transferred to the teleconsulting professional. Currently, the service has two postgraduate dental teleconsultants.

All recommendations suggested by the teleconsultants were based on scientific evidence. The registration of teleconsulting is done by the teleconsultant on a platform developed by TelessaúdeRS-UFRGS and the Ministry of Health. All calls are recorded and subject to audit by the coordination of the service and the Ministry of Health. During the consultation, the applicant was requested to inform the patient's general data (name, age, medical history, dental history, and habits such as tobacco and alcoholic beverages). After then, the applicant described the clinical case and the specific question. At that time, the applicant was expected to send clinical photos or complementary examinations to support the teleconsultant in diagnosis and decision-making. The photos were sent to a WhatsApp™ number of the service, transferred to the TelessaúdeRS-UFRGS' platform, and recorded the files in folder identified by the teleconsultation registration number for further audit.

The possibilities for conducting recommendations by teleconsultation were to (1) maintain the case in PHS, (2) forward the case for face-to-face assistance in specialized care, and (2) forward the case for emergency face-to-face assistance.

## Results

**FIG. 1.**
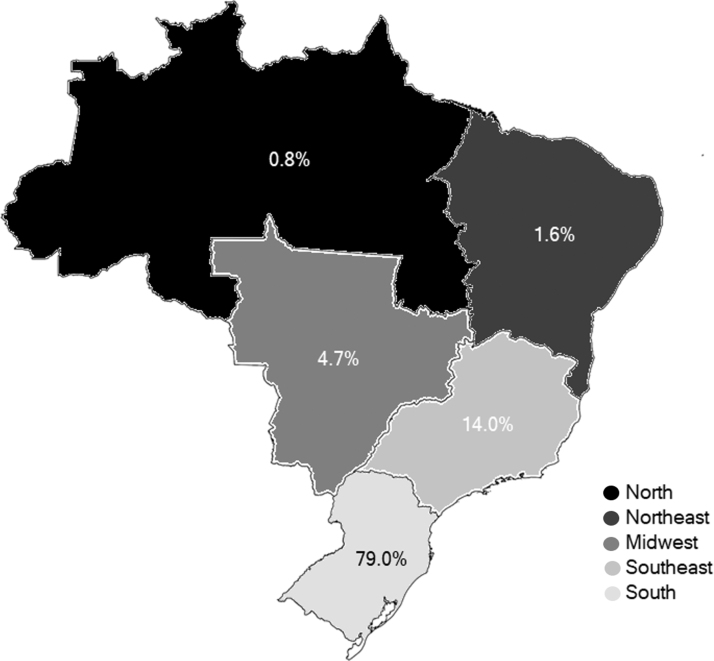
Percentage of teleconsultations by region of the country from May 2018 to July 2019.

We analyzed all the 385 teleconsultations related to oral health from 14 different states and 112 Brazilian municipalities. The requesting professionals were 332 dentists (83.2%) and 53 physicians (13.8%). Most applicants were women 283 (73.5%). The State of Rio Grande do Sul, located in the southern region of the country where the headquarters of the service is, presented most of the requests, 296 (76.9%) (Fig. 1).

Regarding dentistry specialties, most requests 196 (50.9%) related to doubts about the diagnosis and management of oral lesions (oral medicine), followed by questions related to oral maxillofacial surgery 54 (14.4%). Regarding the teleconsultants' recommendation, resolution in PHC was considered possible in 220 (57.1%). The recommendation to referral to the specialist was suggested in 163 (42.3%) cases. Only in 2 (0.5%) cases it was recommended to refer to urgency/emergency care. The teleconsultants' recommendation according to dentistry specialties can be found in Table 1.

**Table 1.** The Teleconsultants' Recommendation According to Dentistry Specialties

Dentistry specialties	Maintained in primary health care	Referred to specialist	Referred to urgency/emergency	Total, *n* (%)
Oral medicine	88	106	2	196 (50.9)
Oral maxillofacial surgery	22	32	0	54 (14)
Public health	28	6	0	34 (8.8)
Patients with special needs	25	6	0	31 (8.1)
Endodontics	22	4	0	26 (6.8)
Temporomandibular disorder	13	6	0	19 (4.9)
Pharmacology	6	0	0	6 (1.6)
Pediatric dentistry	5	1	0	6 (1.6)
Cariology	4	1	0	5 (1.3)
Prosthesis	3	1	0	4 (1)
Periodontics	4	0	0	4 (1)
Total, *n* (%)	220 (57.1)	163 (42.3)	2 (0.5)	385

## Discussion

This article describes preliminary experience of a public countrywide telephone consultation service to support health professionals (dentists and physicians) on questions related to oral health. In the evaluated period, 385 teleconsultations were conducted. In a short period of time, the service was used quite often, indicating that the service is promising and helpful. The teleconsultant can assist PHC professional to identify the degrees of priority for attending the specialist or resolving the case.

Recently, due to the pandemic, the potential of teledentistry has been further explored. In many countries, dental procedures have been canceled and concentrated in major centers.^22^ Therefore, teledentistry has been a good alternative for monitoring patients, limiting unnecessary human contact and displacement to major centers, reducing the risk of spreading COVID-19.^23–25^

Two studies assessed the teleconsultations of the Brazilian Telehealth Program offered in Minas Gerais^9^ and Rio Grande do Sul States.^4^ Those articles comprised both asynchronous (text) and synchronous (videoconference) teleconsultations. Although similar, this report refers only to teleconsultation by telephone, covering applicants from all Brazilian States. Interestingly, Bavaresco et al.^4^ found that teleconsultations to oral health teams induced a 45% reduction in the number of referrals to other levels of care. Some years before, also in Brazil, Rezende et al.^26^ demonstrated that teleconsultations prevented 64.2% of referrals to medical specialists. Those data support the potential of this approach to improve the workflow of the health care system.

Our results highlighted a predilection for Oral Medicine and Oral Surgery specialties, together accounting for 65% of teleconsultations. Oral medicine has already been found as an important source of requests by Paixão et al.^9^ and Bavaresco et al.^4^ This finding could be explained by the perception of dentists that their competence to diagnose oral cancer is low.^27^ Most dentists attribute diagnostic difficulty to limited theory instruction and practical training in oral medicine,^28^ reinforcing that continuing education on this topic is needed. The experience of our team of a service that offers telediagnosis of oral lesions for PHC is in accordance with this finding since referral to specialists was recommended in only 43% of the requests due to their complexity.^5^ Furthermore, teleconsultation can also be considered a tool for improving the ability to diagnose and manage injuries in PHC, since this can be more effective than distance education.^29^

Teleconsultations have been discussed in past years. In general, medical professionals who used teleconsultations did not experience problems daily. However, the low use remains an obstacle to be overcome to explore the full potential of this workforce. Some issues could explain it, such as the lack of stimulus, demand pressure/overload, professional profile, personal interest, and difficulties with the systems.^30^ Recent studies, which evaluated the general dentist perception about teledentistry, showed that although the majority of the dentists had less knowledge about the subject, most of them believe that it can improve oral health, especially in rural populations.^31,32^

Some benefits of the use of teleconsultation in the health care system are unquestionable. Teleconsultation services in medicine have demonstrated to be effective, feasible, and economically viable.^33^ There is a consensus that this strategy may reduce the difficulties of access for the residents of small or remote municipalities where technology, resources, and professionals are scarce. Moreover, those areas usually have young and inexperienced professionals who need additional training. In this sense, teleconsultation services may contribute to improving access to diagnosis and management, as well as continuing education.^34^

The most relevant limitation of this study is the lack of information on the applicants' actions after the teleconsultation. In addition, even though teleconsultation services have the potential to contribute in the workflow process of qualifying primary health services, improvement in health status of the population may not be assured.^33^ Therefore, more studies must be performed to produce more robust evidence about this topic.

## Conclusion

The teleconsultation service by telephone launched by the TelessaúdeRS-UFRGS appears to be a promising tool to support health professionals on oral health issues. Present data support that more than half of the cases (57.1%) were considered possible to be resolved in PHC based on teleconsulting. This service has important potential to improve the effectiveness of primary health services and qualify information for the referral system when face-to-face consultation is necessary. Further studies should explore its impact on the decision to refer cases to specialized services.

## Abbreviations Used

ICT: Information and Communication Technology
NHS: National Health System
PHC: primary health care
TelessaúdeRS-UFRGS: Technical and Scientific Centre for Telehealth of Federal University of Rio Grande do Sul
